# Compensatory and decompensatory alterations in cardiomyocyte Ca^2+^ dynamics in hearts with diastolic dysfunction following aortic banding

**DOI:** 10.1113/JP273879

**Published:** 2017-05-21

**Authors:** Sara Gattoni, Åsmund Treu Røe, Jan Magnus Aronsen, Ivar Sjaastad, William E. Louch, Nicolas P. Smith, Steven A. Niederer

**Affiliations:** ^1^ King's College London, Department of Biomedical Engineering and Imaging Sciences, St Thomas’ Hospital, 4th floor North WingThe Rayne InstituteLondonSE1 7EHUK; ^2^ Institute for Experimental Medical ResearchOslo University Hospital and University of OsloOsloNorway; ^3^ K. G. Jebsen Cardiac Research Centre and Centre for Heart Failure ResearchUniversity of OsloOsloNorway; ^4^ Bjørknes CollegeOsloNorway; ^5^ University of AucklandEngineering School Block 1Level 5, 20 Symonds St.Auckland101New Zealand

**Keywords:** calcium dynamics, cardiac electrophysiology, cell biology, computational modelling, diastolic dysfunction, gene expression, hypertrophy, rat

## Abstract

**Key points:**

- At the cellular level cardiac hypertrophy causes remodelling, leading to changes in ionic channel, pump and exchanger densities and kinetics.
- Previous studies have focused on quantifying changes in channels, pumps and exchangers without quantitatively linking these changes with emergent cellular scale functionality.
- Two biophysical cardiac cell models were created, parameterized and validated and are able to simulate electrophysiology and calcium dynamics in myocytes from control sham operated rats and aortic‐banded rats exhibiting diastolic dysfunction.
- The contribution of each ionic pathway to the calcium kinetics was calculated, identifying the L‐type Ca^2+^ channel and sarco/endoplasmic reticulum Ca^2+^ATPase as the principal regulators of systolic and diastolic Ca^2+^, respectively.
- Results show that the ability to dynamically change systolic Ca^2+^, through changes in expression of key Ca^2+^ modelling protein densities, is drastically reduced following the aortic banding procedure; however the cells are able to compensate Ca^2+^ homeostasis in an efficient way to minimize systolic dysfunction.

**Abstract:**

Elevated left ventricular afterload leads to myocardial hypertrophy, diastolic dysfunction, cellular remodelling and compromised calcium dynamics. At the cellular scale this remodelling of the ionic channels, pumps and exchangers gives rise to changes in the Ca^2+^ transient. However, the relative roles of the underlying subcellular processes and the positive or negative impact of each remodelling mechanism are not fully understood. Biophysical cardiac cell models were created to simulate electrophysiology and calcium dynamics in myocytes from control rats (SHAM) and aortic‐banded rats exhibiting diastolic dysfunction. The model parameters and framework were validated and the fitted parameters demonstrated to be unique for explaining our experimental data. The contribution of each ionic pathway to the calcium kinetics was calculated, identifying the L‐type Ca^2+^ channel (LCC) and the sarco/endoplasmic reticulum Ca^2+^‐ATPase (SERCA) as the principal regulators of systolic and diastolic Ca^2+^, respectively. In the aortic banding model, the sensitivity of systolic Ca^2+^ to LCC density and diastolic Ca^2+^ to SERCA density decreased by 16‐fold and increased by 23%, respectively, relative to the SHAM model. The energy cost of ionic homeostasis is maintained across the two models. The models predict that changes in ionic pathway densities in compensated aortic banding rats maintain Ca^2+^ function and efficiency. The ability to dynamically alter systolic function is significantly diminished, while the capacity to maintain diastolic Ca^2+^ is moderately increased.

## Key points

- At the cellular level cardiac hypertrophy causes remodelling, leading to changes in ionic channel, pump and exchanger densities and kinetics.
- Previous studies have focused on quantifying changes in channels, pumps and exchangers without quantitatively linking these changes with emergent cellular scale functionality.
- Two biophysical cardiac cell models were created, parameterized and validated and are able to simulate electrophysiology and calcium dynamics in myocytes from control sham operated rats and aortic‐banded rats exhibiting diastolic dysfunction.
- The contribution of each ionic pathway to the calcium kinetics was calculated, identifying the L‐type Ca^2+^ channel and sarco/endoplasmic reticulum Ca^2+^ATPase as the principal regulators of systolic and diastolic Ca^2+^, respectively.
- Results show that the ability to dynamically change systolic Ca^2+^, through changes in expression of key Ca^2+^ modelling protein densities, is drastically reduced following the aortic banding procedure; however the cells are able to compensate Ca^2+^ homeostasis in an efficient way to minimize systolic dysfunction.

**Abbreviations**
AB: aortic banding
AP: action potential
Ca_amp_: Calcium amplitude
CaB: background Ca^2+^
DCa: diastolic Ca^2+^ concentration
HF: heart failure
LCC: L‐type Ca^2+^ channel
NCX: Na^+^/Ca^2+^ exchanger
PCa: peak Ca^2+^ concentration
PMCa: plasma membrane Ca^2+^‐ATPase
RyR: ryanodine receptor
SERCA: sarco/endoplasmic reticulum Ca^2+^‐ATPase
SR: sarcoplasmic reticulum

## Introduction

Heart failure (HF) due to left ventricular pressure overload following aortic stenosis or hypertension, remains a serious public health problem, associated with high morbidity and mortality (Drazner, 2011). It is commonly believed that cardiac hypertrophy develops in response to increased left ventricular afterload. This increased pressure, in turn, generates increased load on the myocardial wall and leads to tissue and cellular remodelling. In hypertensive patients these changes initially manifest as diastolic dysfunction, proceeding the development of hypertrophy (Aeschbacher *et al*. 2001) and are associated with increased systolic function in hypertensive animal models (Cingolani *et al*. 2003). At the cellular scale, elevated pressure results in changes in the excitation–contraction system, perturbing the pathways central to the transduction of the electrical signal into mechanical function.

In the heart, the role of Ca^2+^ is fundamentally important for transmitting this electrical timing signal into mechanic contraction. During each heartbeat, an electrical wave of depolarization spreads rapidly through the myocardium inducing an intracellular Ca^2+^ influx through the voltage dependent L‐type Ca^2+^ channels (LCCs) (Hobai & Levi, 1999) triggering the release of more Ca^2+^ from ryanodine receptors (RyRs) in the sarcoplasmic reticulum (SR) (Fabiato & Fabiato, 1979; Sham *et al*. 1995). This resultant increase in cytosolic Ca^2+^ concentration induces contraction at the level of the myofilaments. During relaxation, the diastolic Ca^2+^ level is restored by the sarco/endoplasmic reticulum Ca^2+^‐ATPase (SERCA), driving Ca^2+^ back to the SR and the Na^2+^/Ca^2+^ exchanger (NCX) and the sarcolemma Ca^2+^‐ATPase (PMCa) extruding Ca^2+^ out of the cell.

The use of experimental animal models of left ventricular pressure overload, such as the aortic banding (AB) procedure (Litwin *et al*. 1995; deAlmeida *et al*. 2010), has allowed us to quantitatively characterize changes in Ca^2+^ channels, exchangers and pumps during the development of cardiac hypertrophy in rats. In particular, previous studies have reported the L‐type Ca^2+^ channel flux (Keung, 1989; Xiao & Mcardle, 1994; Momtaz *et al*. 1996; Shorofsky *et al*. 1999), the SERCA Ca^2+^ uptake (Feldman *et al*. 1993; Studer *et al*. 1994; Movsesian & Schwinger, 1998; Miyamoto *et al*. 2000), the SR function (Hasenfuss, 1998; Movsesian & Schwinger, 1998), the peak and amplitude of the calcium transients (Bing *et al*. 1991; Siri *et al*. 1991; Bailey & Houser, 1992; Beuckelmann *et al*. 1992) and the duration of the electrical action potential (AP) (Scamps *et al*. 1990; Beuckelmann *et al*. 1992; Meszaros *et al*. 1996; Wickenden *et al*. 1998; Tomaselli, 1999) to be altered during the progression of hypertrophy. However, instead of investigating integrated cardiac function, these studies have typically focused on the expression of single proteins in isolation by recording measurements from cellular phenotypes without making a direct link to the integrated physiology. These measurements are further confounded by the variation in duration and degree of pressure overload resulting in a spectrum of compensated and decompensated response and limiting comparison across studies. Furthermore, despite this work the complex role of changes in Ca^2+^ handling in cardiac hypertrophy remains poorly understood. Specifically, investigations to date have not sought to distinguish between changes that could be considered to be beneficial (compensatory) or detrimental (decompensatory) for maintaining cardiac function. This is essential for identifying the proteins that derive detrimental mechanisms and manipulate them to maintain their regular levels.

**Figure 1:**
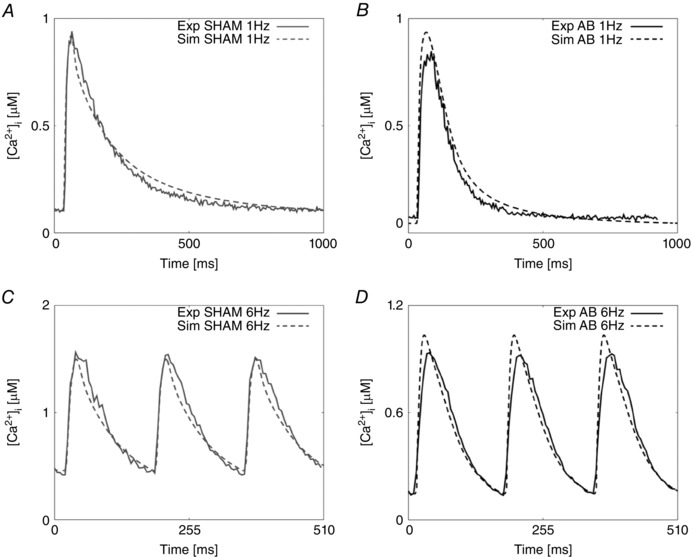
Calcium fitting results *A*, experimental representative Ca^2+^ transient for the SHAM case at 1 Hz (continuous grey line) and Ca^2+^ fitted transient (dashed grey line). *B*, experimental representative Ca^2+^ transient for the AB case at 1 Hz (continuous black line) and Ca^2+^ fitted transient (dashed black line). *C*, experimental representative Ca^2+^ transient for the SHAM case at 6 Hz (continuous grey line) and Ca^2+^ fitted transient (dashed grey line). *D*, experimental representative Ca^2+^ transient for the AB case at 6 Hz (continuous black line) and Ca^2+^ fitted transient (dashed black line).

To address these issues, this study adopts a computational modelling based approach to quantify the relative role of each of the key changes in protein density and kinetics in cardiac myocyte Ca^2+^ phenotypes. To study compensation under elevated ventricular pressure, models are based on experimental recordings from Røe *et al*. (2017), conducted in a focused selection of animals that exhibit maintained systolic function yet diastolic dysfunction 6 weeks post‐AB. A full description of the *in vivo* phenotype including representative MRI pictures and echocardiographic M‐mode recordings is provided in Fig. 1 and Table 1 of Røe *et al*. (2017). Røe *et al*. highlight the role of elevated collagen content and post‐translational modifications in titin that result in increased passive stiffness and organ‐scale diastolic dysfunction. Here we focus on changes in Ca^2+^ that explain the maintenance of Ca^2+^ homeostasis. Firstly, an integrated model of the rat calcium dynamics, fitted to consistent experimental data in both SHAM and AB rats at physiological frequency and temperature is derived. Secondly, to demonstrate that it provides an efficient framework for analysing the mechanisms controlling Ca^2+^ handling in both the SHAM and the AB rat cardiac myocytes, the model parameters and framework are validated. Lastly, a sensitivity analysis is performed to show that the ability of the system to dynamically change systolic Ca^2+^ is drastically reduced in the compensated AB case compared with the SHAM case and the principal compensatory mechanism is an increase in the SERCA protein, which operates to maintain low diastolic Ca^2+^ levels (DCa) at the expenses of systolic Ca^2+^.

**Table 1:** Experimentally measured, representative traces (Rep) and simulated Ca^2+^ metrics in both the SHAM and AB cases at 1 and 6 Hz

Metric	Unit	Type	Frequency	Mean	σ	Rep trace	Simulated
PCa	μm	SHAM	1 Hz	1.000	0.065	0.940	0.915
			6 Hz	1.595	0.111	1.562	1.498
		AB	1 Hz	0.765	0.065	0.8468	0.940
			6 Hz	0.828	0.077	0.9345	1.048
RT_50_	ms	SHAM	1 Hz	137	28.8	135	130
			6 Hz	63	6.4	70	63
		AB	1 Hz	87	15.9	112	121
			6 Hz	57	6.9	80	63
DCa	μm	SHAM	1 Hz	0.100	0	0.096	0.110
			6 Hz	0.374	0.033	0.419	0.458
		AB	1 Hz	0.064	0	0.056	0.045
			6 Hz	0.140	0.011	0.139	0.147
Ca_amp_	μm	SHAM	1 Hz	0.900	0.065	0.844	0.805
			6 Hz	1.221	0.111	1.143	1.490
		AB	1 Hz	0.701	0.065	0.791	0.895
			6 Hz	0.688	0.077	0.795	0.901
APD_90_	ms	SHAM	1 Hz	75	10	72	56
			6 Hz	55	14	63	47
		AB	1 Hz	143	13	147	166
			6 Hz	84	35	126	93

Ca_amp_ is the amplitude of the calcium transient.

## Methods

### Model structure and parameter fitting procedure

The model re‐fitting procedure is described in detail in the Appendix. Briefly, using the rat specific framework developed by Gattoni *et al*. (2016), we parameterized two models representing the SHAM and AB rat cardiomyocytes at both 1 and 6 Hz frequencies at 37°C. In the calcium system we refitted the Na^+^/Ca^2+^ exchanger (NCX), Ca^2+^ pump (PMCa), sarcoplasmic reticulum Ca^2+^‐ATPase (SERCA), L‐type channel (LCC) and background Ca^2+^ channel (CaB). NCX and PMCa densities were constrained by Ca^2+^ transients induced by caffeine exposure following 1 and 6 Hz pacing. These results were then combined with Ca^2+^ transient measurements at 1 and 6 Hz to fit SERCA to the decay of the field stimulated Ca^2+^ transients at 1 and 6 Hz. The Ca^2+^ influx into the cell from the LCCs was fitted using the current–voltage relationship curve. The background Ca^2+^ current was modified to reproduce the peak of the Ca^2+^ transients at 1 and 6 Hz. The remaining Na^+^ and K^+^ currents were constrained using literature data to achieve a prolonged action potential duration commonly found in both human heart failure and in animal models of cardiac hypertrophy. The effect of EGTA in patched pipettes was modelled by setting [EGTA] = 0 mm and 0.6 mm in Ca^2+^ transient and AP experiment simulations, respectively.

### Validation

To test the resulting parameters for both the SHAM and AB rat models we performed a comprehensive model validation study. A schematic representation of the validation process is shown in Fig. 9 of the Appendix. As outlined in ‘Model structure and parameter fitting procedure’ above, we created parameter sets that represent the kinetics of SERCA, LCC and NCX in the SHAM and the AB state. To validate these parameter sets we made nine test models starting with the SHAM model. The test models were developed to observe the effects of single or groups of protein changes on the Ca^2+^ dynamics. In three test models we introduced the AB NCX, LCC or SERCA model with all other parameters kept at the SHAM values (LCC, NCX, SERCA). In three additional models we introduced pairs of LCC, NCX and SERCA AB models into the SHAM rat model (LCC–SERCA, NCX–SERCA, LCC–NCX) and finally we introduced NCX, LCC and SERCA AB models into the SHAM model (LCC–SERCA–NCX). We created two further models. The first started with the SHAM model and included SERCA, NCX and LCC AB models with the changes in K^+^ and Na^+^ channels from the AB model (LCC–SERCA–NCX–K–Na). The second was the same as the previous model but also included the background Ca^2+^ current parameters from the parameters used in the AB model. These nine models were simulated at 1 and 6 Hz giving a total of 18 simulated Ca^2+^ and AP transients. For each of these 18 models we performed graded changes in 17 of the main protein parameters regulating the electrophysiology currents: *g*_SERCA_: conductivity of the SERCA pump; *K*_SERCA_: half‐inactivation constant of the SERCA pump; *J*_L_: permeability of the L‐type channel; *J*_R_: permeability of the RyR; *g*_CaB_: conductivity of the background Ca^2+^ current; *g*_SRl_: conductivity of the SR leak; *g*_t_: conductivity of the transient outward potassium current; *g*_NCX_: conductivity of the Na^+^/Ca^2+^ exchanger; *g*_pCa_: conductivity of the Ca^2+^ pump; *g*_BK_: conductivity of the background K^+^ current; *g*_BNa_: conductivity of the background Na^+^ current; *g*_K1_: conductivity of the inward potassium current; *g*_Na_: conductivity of the Na^+^ current; *g*_ss_: conductivity of the steady state potassium current; NaK_max_: conductivity of the Na^+^/K^+^ exchanger; *B*_CMDN_: calmodulin concentration; and *B*_TRPN_: troponin concentration. Each parameter *x* was perturbed in the range log_10_(*x*_new_) ∈ [−1,1], where *x*_new_ is the parameter value used for a generic simulation and *x*_Base_ is the fitted parameter value (in this way −1 corresponds to 10% of the control parameter and 1 corresponds to a 10‐fold increase). All developed models, representing 5814 parameter combinations were run to their limit cycle. For each model we identified how many of experimental phenotypes (peak calcium concentration (PCa), DCa, relaxation time at 50% decay (RT_50_) and time to peak Ca^2+^ (*T*_peak_)) the model matched and we ranked models based on this number (1–4). A parameter combination is defined ‘viable’ if the considered simulated phenotype value falls within a 20% range of the experimental phenotype value (see Appendix, ‘Validation method and tables’).

### Sensitivity analysis

The sensitivity of four calcium transient phenotypes, PCa, DCa, RT_50_ and *T*_peak_, were calculated for 11 protein densities: *g*_SERCA_, *K*_SERCA_, *g*_CaB_, *g*_t_, *g*_K1_, *g*_Na_, *g*_ss_, *V*_L_ (potential when half LCC channels are open), *K*_L_ (concentration at LCC inactivation), *J*_L_, and *g*_NCX_. The sensitivity values were calculated with a 1% change in the protein density for both the SHAM and AB models’ proteins at 6 Hz, using the equation:

$$S_{i,j}=\frac{dP_{i}}{dp_{j}}\cdot \frac{p_{j}}{P_{i}}=\frac{P\left(p_{1}\right)-P\left(p_{0}\right)}{p_{1}-p_{0}}\cdot \frac{p_{0}}{P(p_{0})}$$

where *p*_1_ is the new parameter (increased by 1% from the fitted parameter), *p*_0_ is the fitted parameter, $P(p_{1})$ is the tested phenotype value evaluated in the new parameter *p*_1_, and $P(p_{0})$ is the tested phenotype value evaluated in the fitted parameter *p*_0_.

## Results

### Fitting models of SHAM and AB at 1 and 6 Hz

**Figure 2:**
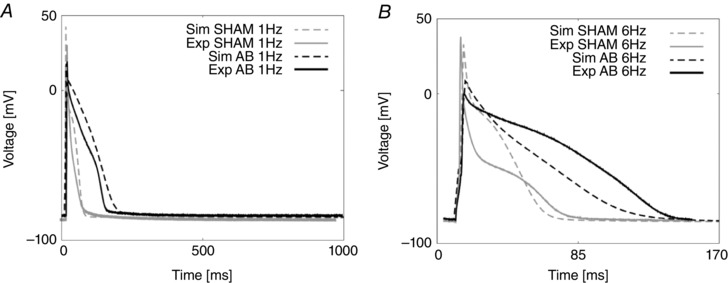
AP fitting results *A*, experimental and simulated AP traces for the SHAM case (grey) and the AB case (black) at 1 Hz. *B*, experimental and simulated AP traces for the SHAM case (grey) and the AB case (black) at 6 Hz.

Fig. 1 and 2 show the experimental and simulated Ca^2+^ and AP traces for the SHAM and AB models at 1 and 6 Hz. At 1 Hz, simulated DCa and RT_50_ in the AB model were reduced by 59% and 7%, respectively, compared with the DCa and RT_50_ simulated values in the SHAM model and consistent with a decrease of 42% and 17%, respectively, observed experimentally. At the same frequency, simulated PCa was increased by 3% although experimental measurement revealed a 7% decrease. Simulated SR Ca^2+^ content in the AB model at 1 Hz was increased by 28% compared with the simulated value in the SHAM case and compatible with increased SERCA activity in both experimental and simulated measurements.

At 6 Hz, simulated DCa in the AB model decreased by 68%, consistent with the experimentally observed 67% decrease. Simulated RT_50_ in the SHAM model remained unchanged in the AB model with a value of 63 ms, falling within mean experimental measurements of 63 ± 6.4 and 57 ± 7 ms in the SHAM and AB cases, respectively. At the same frequency, simulated PCa in the AB model was decreased by 30% with respect to the simulated value in SHAM model, comparable to a 40% decrease in the experimental data. Simulated SR Ca^2+^ content in the AB model at 6 Hz was increased by 61% compared with the simulated value in the SHAM model, due to increased SERCA activity. Key Ca^2+^ characteristics are summarized in Table 1.

We predicted the proportion of Ca^2+^ extrusion through SERCA, NCX and PCA by evaluating their simulated integrals during one cycle. Results in the SHAM case reveal a ratio of 86.6%, 10% and 3.4%, respectively, at 1 Hz, and 94%, 5% and 1%, respectively, at 6 Hz. Results in the AB case reveal a proportion of 92%, 6.3% and 1.7%, respectively, at 1 Hz and 96.4%, 2.8% and 0.8%, respectively, at 6 Hz. Prolonged duration of the electrical AP is shown in Fig. 2. At the lower pacing frequency of 1 Hz, we predicted pronounced prolongation in late repolarization in the AB model compared with the SHAM model, with the time to 90% repolarization (APD_90_) increased by 66%, comparable to an increase of 50% in the experimental data. At the higher pacing frequency of 6 Hz, repolarization was faster compared with the same model at 1 Hz and we predicted APD_90_ to be increased by 50%, comparable with a 50% increase in the experimental data.

### Validation

Our model validation tables are represented in Figs 10, 11, 12, 13, 14, 15, 16 in the Appendix. Without considering the trivial viable AB cases, our simulations reveal approximately 28% and 54% viable parameter combinations, respectively, at 1 and 6 Hz, when considering a single calcium phenotype, 6% and 24% when considering two calcium phenotypes and less than 1% (38 combinations) and 3% when considering three calcium phenotypes. When considering all four calcium phenotypes in Fig. 16 of the Appendix, less than 1% (5 combinations) and less than 1% (13 combinations) of the viable parameter combinations are able to match the experimental recordings, respectively, at 1 and 6 Hz. Among these combinations, only four parameter combinations are able to match all four phenotypes at both pacing frequencies and correspond to an increase in Ca^2+^ pump conductivity *g*_PCa_ $\;\varepsilon \;[1.256\;\times \;10^{-5},\;1.990\;\times \;\;10^{-5}]$ and a decrease in the background current conductivity *g*_CaB_ $\varepsilon \;[0.5024\;\times \;10^{-8},\;1.0024\;\times \;10^{-8}]$. The resulting simulated PCa, DCa, RT_50_, *T*_peak_ and Calcium amplitude (Ca_amp_) values for those four parameter combinations produce very similar results, as shown in Table 2. The value we chose for the background Ca^2+^ conductivity parameter *g*_CaB_ = $0.6\;\times \;10^{-8}\;$falls within the acceptable range.

**Table 2:** PCa, DCa, RT_50_, *T*_peak_ and Ca_amp_ values obtained for the four acceptable parameter combinations

		PCa (μm)	RT_50_ (ms)	DCa (μm)	*T*_peak_ (ms)	Ca_amp_ (μm)
g pCa =1.256×10−5	1 Hz	0.998	111	0.052	14	0.946
	6 Hz	1.014	62	0.136	21	0.878
g pCa =1.990×10−5	1 Hz	0.845	115	0.047	41	0.798
	6 Hz	0.923	61	0.125	26	0.798
g CaB =0.502×10−8	1 Hz	0.920	124	0.044	38	0.876
	6 Hz	1.042	67	0.146	21	0.896
g CaB =1.002×10−8	1 Hz	1.014	121	0.049	34	0.964
	6 Hz	1.071	68	0.151	20	0.919

### Energy cost

To determine if the changes in Ca^2+^ kinetics are associated with a change in the energetic cost of maintaining ionic homeostasis, we calculated the ATP consumption over one cardiac cycle. For each ion transporter, taking into account its specific stoichiometry (SERCA: 2 Ca^2+^ : 1 ATP; PMCa: 1 Ca^2+^ : 1 ATP; and Na^+^/K^+^: 3 Na^+^ : 1 ATP), we calculated the ATP consumption per cardiac cycle as the integral of the rate of ATP consumption over each cardiac cycle.

Comparison of ATP consumption per cardiac cycle showed no relevant changes, with total values of 31.15 and 32.55 μm per beat respectively in the SHAM and AB cases at 6 Hz. ATP consumption by SERCA, Na^+^/K^+^ and PMCa at 6 Hz shows values of 24.1, 4.15 and 2.9 μm, respectively, in the SHAM case and 27.4, 2.92 and 2.23 μm, respectively, in the AB case.

### Sensitivity analysis

**Figure 3:**
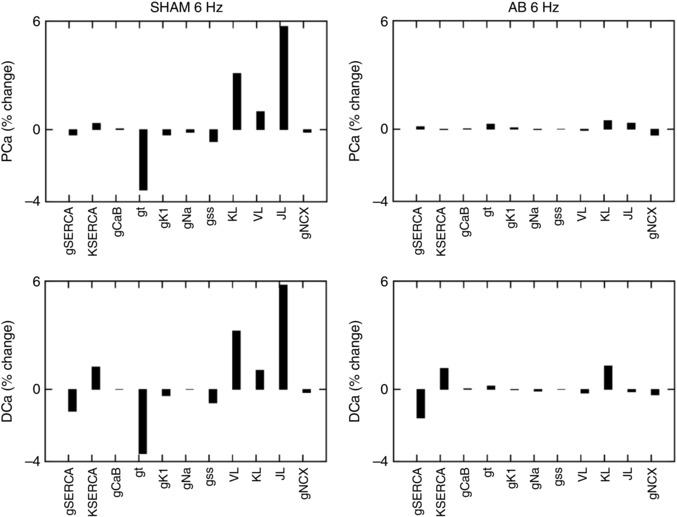
Sensitivity analysis on PCa and DCa at 1% Percentage changes in peak [Ca^2+^]i (PCa) and diastolic [Ca^2+^]i (DCa) in the SHAM case (left panels) and AB case (right panels). Sensitivity was studied by performing a 1% increase of the most important protein parameters changing from SHAM to AB: *g*_SERCA_, *K*_SERCA_, *g*_CaB_, *g*_t_, *g*_K1_, *g*_Na_, *g*_ss_, *J*_L_ and *g*_NCX_. In the case of the sensitivity value being positive, an increase in the parameter will lead to an increase in the output. In the case of the sensitivity value being negative, an increase in the parameter will lead to a decrease in the output.

**Figure 4:**
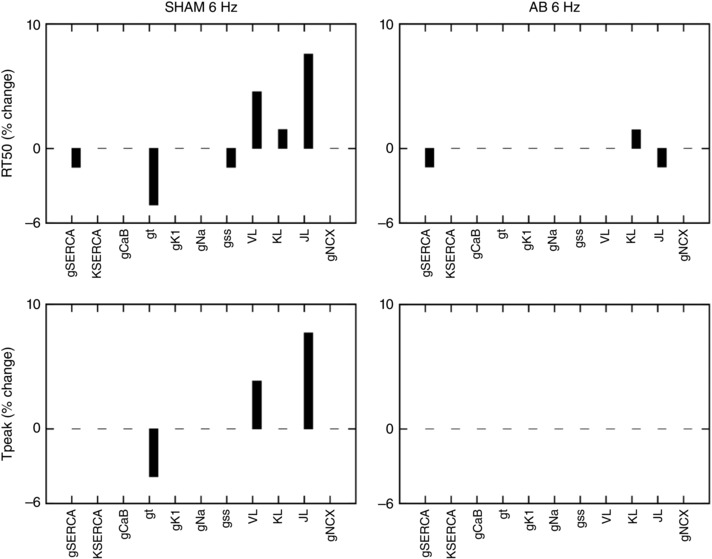
Sensitivity analysis on RT_50_ and *T*_peak_ at 1% Percentage change in relaxation time at 50% (RT_50_) and time to peak [Ca^2+^]_i_ (*T*_PEAK_) in the SHAM case (left panels) and AB case (right panels). Sensitivity was studied by performing a 1% increase of the most important protein parameters changing from SHAM to AB: *g*_SERCA_, *K*_SERCA_, *g*_CaB_, *g*_t_, *g*_K1_, *g*_Na_, *g*_ss_, *J*_L_ and *g*_NCX_. In the case of the sensitivity value being positive, an increase in the parameter will lead to an increase in the output. In the case of the sensitivity value being negative, an increase in the parameter will lead to a decrease in the output.

To estimate the importance of different proteins during the progression from SHAM to AB and to distinguish between compensatory and decompensatory mechanisms, a sensitivity analysis was performed. The sensitivity values are shown in Table 3 for both the SHAM and AB cases. In Figs 3 and 4, we compared the effects of the sensitivity analysis in the SHAM model with that in the AB model.

**Table 3:** Sensitivity analysis for the SHAM and AB models

		PCa		DCa		RT_50_		*T*_peak_	
Parameter	Description	SHAM	AB	SHAM	AB	SHAM	AB	SHAM	AB
*g*_SERCA_	Maximum pump rate of SERCA	−0.301	0.138	−1.217	−1.588	−1.515	−1.493	0	0
*K*_SERCA_	Half saturation of SERCA	0.351	−0.029	1.246	1.173	0	0	0	0
*g*_CaB_	Conductance of background Ca^2+^	0.050	0.024	0.0014	0.0357	0	0	0	0
*g*_t_	Conductance of transient outward K^+^	−3.355	0.280	−3.563	0.1959	−4.545	0	−3.846	0
*g*_K1_	Conductance inward rectifying K^+^	−0.304	0.080	−0.352	−0.0211	0	0	0	0
*g*_Na_	Conductance of inward Na^+^	−0.157	−0.027	8.7 × 10^–5^	−0.098	0	0	0	0
*g*_ss_	Conductance of steady state K^+^	−0.664	0.004	−0.744	−0.003	−1.515	0	0	0
*V*_L_	Potential when half LCC channels are open	−0.664	−0.087	3.242	−0.217	4.546	0	3.846	0
*K*_L_	Concentration at LCC inactivation	3.111	0.481	1.061	1.299	1.515	1.492	0	0
*J*_L_	Permeability of a single LCC	5.706	0.342	5.777	−0.132	7.576	−1.492	7.692	0
*g*_NCX_	Pump rate of NCX	−0.151	−0.335	−0.169	−0.302	0	0	0	0

Sensitivity values S_*i,j*_, where *i* are the phenotypes and *j* are the tested parameters. In the case of the sensitivity being positive, an increase in the parameter will lead to an increase in the output. In the case of the sensitivity being negative, an increase in the parameter will lead to a decrease in the output.

The sensitivity analysis for the SHAM model at 6 Hz in Fig. 3, reveals the dominant role of the L‐type channel and transient outward K^+^ current parameter changes on all the studied Ca^2+^ phenotypes. In Table 3 we show that in the SHAM case a 1% increase in *J*_L_ (LCC permeability) leads to a 5.7% increase in PCa, 5.8% increase in DCa, 7.6% increase in RT_50_ and 7.7% increase in *T*_peak_. Conversely, a 1% increase in *g*_t_ (*I*_to_ conductance) leads to a 3.3% decrease in PCa, 3.6% decrease in DCa, 4.5% decrease in RT_50_ and 3.8% decrease in *T*_peak_.

In the AB model at 6 Hz in Fig. 4 we see a reduced magnitude in the sensitivity for almost all parameters. In Table 3 we show that none of the tested parameters has a dominant role on PCa or *T*_peak_ in the AB model. A 1% increase in all tested parameters leads to less than 0.5% change in PCa and no change in *T*_peak_. A dominant role of *g*_SERCA_ is visible on DCa where a 1% increase in this parameters produces a 1.6% decrease in DCa. *J*_L_ and *g*_SERCA_ have major effects on RT_50_ where a 1% increase in both those parameters leads to a 1.5% decrease in RT_50_.

### Backward and forward projections

**Figure 5:**
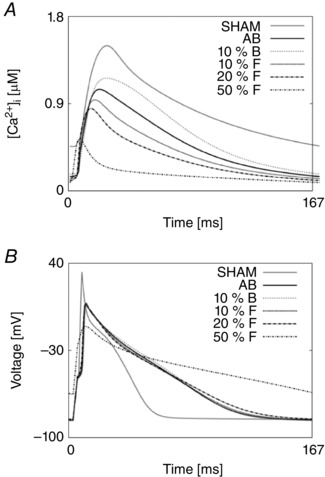
Backward and forward projections Starting from the AB model, we performed a 10% backward change in all parameters (towards the SHAM case) and a 10%, 20% and 50% forward change in all parameters (towards the AB case). *A*, simulated changes in the calcium transient. *B*, simulated changes in the AP.

To observe the joint effect of all altered subcellular proteins during the transition from SHAM to the final AB state, in Fig. 5 we simulated backward and forward projections on the Ca^2+^ transient and AP. Starting from the AB model, we performed a 10% backward linear change in all parameters (towards the SHAM case) and a 10%, 20% and 50% linear change in all parameters forward towards the AB case. In Fig. 5, starting from the AB case and moving forward we see a significantly reduced PCa for only minor changes in DCa, suggesting that the system as a whole is compensating by reducing DCa at the expenses of PCa.

## Discussion

Alterations of excitation–contraction coupling, resulting from altered proteins in calcium homeostasis, are thought to play a critical role in heart failure (Balke & Shorofsky, 1998; Hasenfuss, 1998; Wasserstrom *et al*. 2009; Luo & Anderson, 2013). As many of the cellular mechanisms responsible for the development of hypertrophy and its progression to HF have not yet been identified, a better understanding of the primary mechanisms involved in this process has the potential to facilitate the development of new therapeutic techniques and ultimately guide prevention.

We have developed a biophysical model of rat ventricular cardiac myocyte electrophysiology and Ca^2+^ dynamics, parameterized at physiological temperature and pacing frequency and capable of capturing Ca^2+^ and AP responses in both the SHAM and AB conditions. To both test the relevance of our model and underpin the conclusions derived from it, we also performed a comprehensive validation exercise. To reveal the main Ca^2+^ pathways responsible for the changes in the transition from SHAM to AB and distinguish between compensatory and decompensatory mechanisms, we have performed a sensitivity analysis of the responses of Ca^2+^ phenotypes to changes in the key [Ca^2+^]_i_ proteins during the progression of diastolic dysfunction, 6 weeks post‐AB, in rats. Experimentally, the animals do not show signs of HF. These results show that the ability to dynamically change systolic Ca^2+^, through changes in expression of key Ca^2+^ modelling protein densities, is drastically reduced following the AB procedure. However the cells are able to compensate Ca^2+^ homeostasis and efficiently minimize diastolic dysfunction caused by passive stiffening of the myocardium (Røe *et al*. 2017).

### Phenotype and current changes following AB

Our model‐based findings are consistent with the major part of the experimental literature. In the AB model we reported a decrease in PCa and Ca^2+^ amplitude compared with the SHAM case at 6 Hz. This finding is consistent with both the experimental data in this study and the work done by Moore *et al*. (1991) who showed the peak and amplitude of the Ca^2+^ transient to decrease in rat hypertrophied cardiac myocytes following occlusion of the left renal artery. Both our experimental measurements and our simulations showed reduced DCa at 6 Hz in the AB case compared with the SHAM, consistent with results of Bing *et al*. (1991) in spontaneously hypertensive rats. Following the AB procedure, the RT_50_ value was shown to decrease in both our experimental and our simulated results at 6 Hz.

In our experiments we reported an increase in the L‐type Ca^2+^, SERCA and NCX channels following the AB procedure. Consistent with the experimental measurements and the work by Xiao & Mcardle (1994) and Keung (1989), who reported an increase in the L‐type Ca^2+^ channel density for moderate hypertrophy cases, in the AB model we showed an increase in L‐type Ca^2+^ channel density.

We simulated an increase in SERCA activity following AB, concordant with the experimental measurements in this work. Many studies have reported decreased SERCA activity or gene expression in hypertrophy and HF (Studer *et al*. 1994; Miyamoto *et al*. 2000), an observation that is not replicated in our work. Decreased SERCA expression and function are a consistent finding in severe HF and are thought to be responsible for prolonged relaxation leading to failure. Movsesian & Schwinger (1998) found that the levels of SERCA and phospholamban are comparable in normal and failing human myocardium and Feldman *et al*. (1993) reported decreased Ca^2+^‐ATPase mRNA in rats 20 weeks after AB with no change in the early stages (8 weeks after AB) suggesting that the decrease in Ca^2+^‐ATPase mRNA levels may be a marker of the transition from compensatory hypertrophy to failure in rats.

In our simulation study, we reported increased NCX activity following AB, consistent with the experimental data in our work and the majority of results in the literature (Studer *et al*. 1994; Reinecke *et al*. 1996) while others showed decreased NCX activity (Hanf *et al*. 1988). In the AB experiments we showed a prolongation of the electrical AP compared with the SHAM case. Human and animal models of hypertrophy and failure previously showed a prolongation of the electrical AP (Scamps *et al*. 1990; Beuckelmann *et al*. 1992; Meszaros *et al*. 1996; Wickenden *et al*. 1998; Tomaselli, 1999), thought to be caused by a significantly reduced density of the transient outward potassium current (*I*_to_) (Tomita *et al*. 1994; Meszaros *et al*. 1996; Wickenden *et al*. 1998; Tomaselli, 1999), an observation that is captured by the model. Despite these changes, in the AB model we predicted no change in the ATP consumption at 6 Hz, compared with the SHAM model. Consistent with these findings, previous work in mice developed by Li *et al*. (2012) showed the ATP consumption per cardiac cycle to be maintained in the early stages of HF. Overall results in Fig. 5, suggest that the model is acting to reduce DCa although PCa is drastically reduced.

### Validation

In our work, we have implemented our validation method by exploring 5814 permutations of parameters to show that model components, in combination with parameters in our model, provide a robust representation of the effects of AB in cardiac myocytes. In Figs. 10 and 13 of the Appendix we observe a very large number of parameter combinations falling inside a 20% variability of single metrics in both the 1 Hz and the 6 Hz cases (positive cases in grey). Positive cases are spread among different models in the PCa, RT_50_ and *T*_peak_ tables at 1 Hz while we see clustered columns of positive cases in the DCa table, corresponding to all models that include SERCA changes and thus suggesting that SERCA is the principle mechanism responsible for decreased DCa in the AB model at 1 Hz frequency. The same analysis can be done for the single metrics at 6 Hz although we report mainly positive cases in the RT_50_ and *T*_peak_ tables and the DCa table shows clustered positive cases only when SERCA changes are coupled with NCX changes, thus suggesting a possible secondary role of NCX in decreasing DCa in the AB model.

When looking at metric couples and triplets in Figs 11, 12, 14 and 15 in the Appendix, we see a drastically reduced number of positive cases. In particular, when observing the effects of changes in all metrics together in Fig. 16, removing the AB cases that clearly fall in the 20% ranges, we see only three grey boxes at 1 Hz and four grey boxes at 6 Hz. Among these combinations, only four parameter combinations are able to match all four phenotypes at both pacing frequencies. This method demonstrates that our choice of parameters provides a robust representation of the effects of AB in cardiac myocytes, validating the parameter set. Furthermore, as none of the parameter combinations gave positive results for all Ca^2+^ metrics at 1 and 6 Hz, when only including the data‐driven Ca^2+^ changes of LCC, NCX and SERCA, we conclude that reduced Na^+^ and K^+^ currents are not solely responsible for the increased APD; however, they are implicated in Ca^2+^ changes in the AB models.

### Reduced ability to dynamically change Ca^2+^ following AB

The sensitivity analysis shows that the L‐type channel (*J*_L_) and transient outward K^+^ (*g*_t_) conductivities are important in the SHAM model in that increased *J*_L_ leads to increased PCa, DCa, RT_50_ and *T*_peak_ while increased *g*_t_ leads to decreased PCa, DCa, RT_50_ and *T*_peak_. The magnitude of this sensitivity is drastically reduced following AB. The ability to dynamically change systolic Ca^2+^ is an important property of cardiac myocytes in so much that it enables regulation of the strength of cardiac contraction and thus plays a role in the regulation of blood supply throughout the body across a wide range of different conditions, including exercise. In this context, reduced magnitude of the sensitivity following AB is a clear sign of diminishing function, in the sense that the system is less responsive to changes in the AB state compared with the SHAM state. Despite numerous investigations, the role of L‐type Ca^2+^ channels in the pathogenesis of cardiac hypertrophy and HF remains controversial (Shorofsky *et al*. 1998). Studies in rats have reported unchanged (Scamps *et al*. 1990; Shorofsky *et al*. 1998) increased (Keung, 1989; Xiao & Mcardle, 1994) or decreased L‐type channel density (Santos *et al*. 1995). In particular, the effects of cardiac hypertrophy on cardiac L‐type Ca^2+^ currents seem to depend on the progression of the disease. Xiao & Mcardle (1994) observed an increase in L‐type Ca^2+^ current density in spontaneously hypertensive rat myocytes at 10 weeks, whereas other investigators predicted no change in current density in rat cells at a later age (Brooksby *et al*. 1993; Cerbai *et al*. 1994). Our sensitivity analysis shows a drastic reduction in systolic Ca^2+^ sensitivity to L‐type Ca^2+^ from SHAM to AB, consistent with the idea of a change in the L‐type channel behaviour during disease progression (Santos *et al*. 1995). Effects of protein changes on DCa are also potential markers of diminishing function. It is worth noting that SERCA is an exception to this trend, being the only mechanism able to reduce DCa and amplify its effect following AB. This result not only confirms the importance of SERCA in maintaining the DCa level at the expenses of systolic Ca^2+^ but highlights its unique role as a possible marker of the transition from compensatory hypertrophy to failure in rats, as postulated previously by Feldman *et al*. (1993). In this context it is particularly interesting to observe the direction of the effects. Specifically, if a protein continues to increase (or decrease) the question is, will its effect on a given Ca^2+^ phenotype reverse? Assuming that changes in phenotypes are monotonic when increasing or decreasing parameters, compensatory effects will be concordant in the SHAM and AB cases (both positive and negative) while decompensatory effects will be discordant in the SHAM and AB cases (one positive, one negative). In this way, we assume that there is a protein direction of change at SHAM that will improve function. However, if the sensitivity is discordant then this means that this given protein is having the opposite effect on Ca^2+^ function in AB compared with SHAM animals, thereby indicating a transition where changes in a protein that were initially beneficial are now detrimental. In our sensitivity analysis, all the SERCA, inward rectifying K^+^ current, steady state K^+^ current and transient outward K^+^ current parameters show decompensatory effects on peak Ca^2+^ while the L‐type Ca^2+^ current, the inward Na^+^ current and the transient outward K^+^ current parameters show decompensatory effects on diastolic Ca^2+^. Focusing on the magnitude of the sensitivity, the L‐type Ca^2+^ channel and the transient outward K^+^ channel are the most significant pathways regulating PCa while SERCA is the most significant pathway regulating DCa. The transient outward K^+^ channel shows decompensatory effects on both PCa and DCa while the L‐type Ca^2+^ current reveals a compensatory effect on PCa and a decompensatory effect on DCa. SERCA changes reveal a compensatory effect on DCa and a decompensatory effect on PCa. Overall, results following AB reveal the major compensatory effect of SERCA on DCa at the expenses of PCa, highlighting the trend of the system to reduce DCa in order to maintain a physiological Ca^2+^ balance as a response to AB (see Fig. 5). Furthermore, Fig. 5 reveals non‐significant changes in AP with the backward and forward projections at 10% and 20% levels, suggesting that the AP is not affecting the calcium transient. An increase in the resting membrane potential and a prolongation of the AP is observed at a 50% forward change in parameters.

### Conclusions

In our work we have concluded that in rats where the systolic properties of the ventricle are not altered 6 weeks after the AB procedure, the cellular system reveals the first signs of disease as its ability to dynamically change systolic and diastolic Ca^2+^ is drastically reduced. Following compensated AB, SERCA is the only major Ca^2+^ handling protein that maintains its principal role in ensuring low DCa levels, confirming its unique role as a possible marker of the transition from compensatory hypertrophy to failure in rats.

## Appendix

In our work we have re‐parameterized the electrophysiology model developed by Gattoni *et al*. (2016). Here we describe the model parameter re‐fitting procedure for all the fitted channels within the control (SHAM) model and the aortic‐banding (AB) model, we show the validation procedure and results, and we highlight the main limitations of the work.

### Parameter refitting procedure

The parameter fitting procedure adopted to re‐parameterize the electrophysiology framework developed in Gattoni *et al*. (2016) is described in detail. A summary of all the fitted parameters within the SHAM and AB models is shown in Tables 4 and 5, respectively.

**Table 4:** Summary of all the fitted parameters in the SHAM model

Parameter	Unit	Definition
g NCX =0.0234×10−3	μm ^3^ ms^–1^	Pump rate of NCX
g PMCa =0.005×10−3	μm ^3^ ms^–1^	Maximum pump rate of sarcolemmal pump
K PMCa =0.35×10−3	mm	Half saturation of sarcolemmal pump
g SERCA 1=0.235×10−3	μm ^3^ ms^–1^	Maximum pump rate of SERCA at 1 Hz
K SERCA 1=0.497×10−3	mm	Half saturation of SERCA at 1 Hz
g SERCA 6=0.51×10−3	μm ^3^ ms^–1^	Maximum pump rate of SERCA at 6 Hz
K SERCA 6=0.69×10−3	mm	Half saturation of SERCA at 6 Hz
$\;J_{L}=\;8\times 10^{-4}$	μm ^3^ ms^–1^	Permeability of single LCC
$\;K_{L}=\;0.00038$	mm	Concentration at inactivation LCC
$\;V_{L}=\;-9$	mV	Potential when half LCC open
δ VL =7	mV	Width of opening potential
$\varphi_{L}\;=\;11.5$	Unitless	Proportion of time closed in open mode LCC
$\;\tau_{L}=\;1550$	ms	Inactivation time
$\;g_{D}=\;0.1$	μm ^3^ ms^–1^	Permeability of the leak current from the dyadic space
g SRL =1×10−6	ms^–1^	Rate of leak from the SR to the cytosol
g CaB =2×10−8	mm mV^–1^ ms^–1^	Background Ca^2+^ current rate
$\;g_{t}=\;2\times 10^{-5}$	mS	Permeability of the transient outward potassium current
g ss =1.3×10−5	mS	Permeability of the steady state potassium current
$\;g_{K1}=\;4\times 10^{-5}$	mS	Permeability of the inward rectifying current
g Na =0.0007	mS	Permeability of the inward sodium current

**Table 5:** Summary of all the fitted parameters in the AB model

AB fitted parameters		
Parameter	Unit	Definition
g NCX =0.0456×10−3	μm ^3^ ms^–1^	Pump rate of NCX
g PMCa =0.005×10−3	μm ^3^ ms^–1^	Maximum pump rate of sarcolemmal pump
K PMCa =0.35×10−3	mm	Half‐saturation of sarcolemmal pump
g SERCA 1=0.49×10−3	μm ^3^ ms^–1^	Maximum pump rate of SERCA at 1 Hz
K SERCA 1=0.44×10−3	mm	Half‐saturation of SERCA at 1 Hz
g SERCA 6=0.49×10−3	μm ^3^ ms^–1^	Maximum pump rate of SERCA at 6 Hz
K SERCA 6=0.25×10−3	mm	Half saturation of SERCA at 6 Hz
$\;J_{L}=\;12\;\times \;10^{-4}$	μm ^3^ ms^–1^	Permeability of single LCC
$\;K_{L}=\;0.00016$	mm	Concentration at inactivation LCC
$\;V_{L}=\;-13$	mV	Potential when half LCC open
δ VL =7	mV	Width of opening potential
$\varphi_{L}\;=\;11.5$	Unitless	Proportion of time closed in open mode LCC
$\;\tau_{L}=\;1550$	ms	Inactivation time
$\;g_{D}=\;0.1$	μm ^3^ ms^–1^	Permeability of the leak current from the dyadic space
g SRL =1×10−6	ms^–1^	Rate of leak from the SR to the cytosol
g CaB =6×10−9	mm mV^–1^ ms^–1^	Background Ca^2+^ current rate
$\;g_{t}=\;1.4\times 10^{-5}$	mS	Permeability of the transient outward potassium current
g ss =1.0×10−6	mS	Permeability of the steady state potassium current
$\;g_{K1}=\;1.5\times 10^{-5}$	mS	Permeability of the inward rectifying current
g Na =0.0002	mS	Permeability of the inward sodium current

Temperature was set to 310 K or 37**°**C to reflect experimental conditions that mimicked physiological temperatures. The parameters for capacitance, volumes, buffering parameters and intracellular and extracellular concentrations were kept constant from the model of a healthy myocyte (Gattoni *et al*. 2016), as these were not measured directly and there were no reports indicating that these values change under AB conditions.

**Figure 6:**
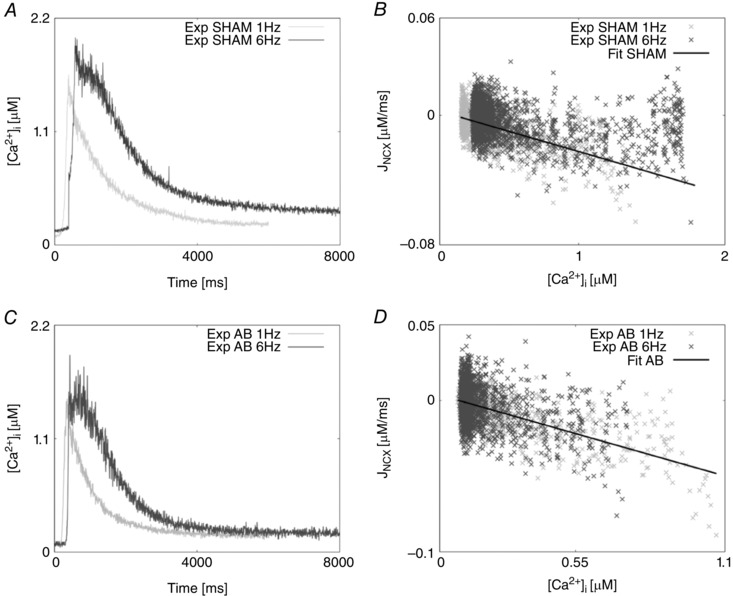
Caffeine transients and NCX fitting results *A*, experimentally measured caffeine‐induced transients for the SHAM case recorded at 1 Hz (light grey) and 6 Hz (dark grey). *B*, NCX flux (*J*_NCX_) as a function of internal Ca^2+^ in the SHAM case at 1 Hz (light grey) and 6 Hz (dark grey). The fitting is represented by a straight line (black). *C*, experimentally measured caffeine‐induced transients for the AB case recorded at 1 Hz (light grey) and 6 Hz (dark grey). *D*, NCX flux (*J*_NCX_) as a function of internal Ca^2+^ in the AB case at 1 Hz (light grey) and 6 Hz (dark grey). The fitting is represented by a straight line (black).

**Figure 7:**
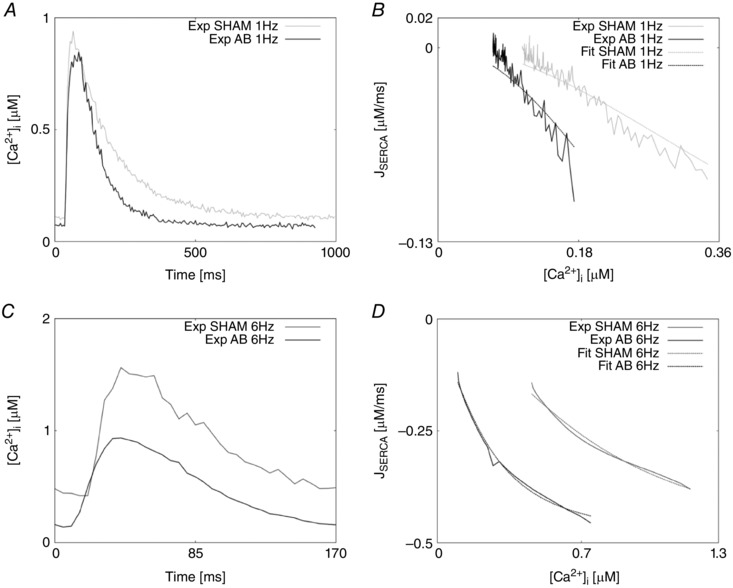
Calcium transients and SERCA fitting results *A*, experimentally measured Ca^2+^ transients recorded at 1 Hz in the SHAM case (grey) and AB case (black). *B*, SERCA flux (*J*_SERCA_) as a function of internal Ca^2+^ at 1 Hz in the SHAM case (continuous grey line) and AB case (continuous black line). Fittings at 1 Hz are represented in the SHAM case (dashed grey line) and AB case (dashed black line). *C*, experimentally measured Ca^2+^ transients recorded at 6 Hz in the SHAM case (grey) and AB case (black). *D*, SERCA flux (*J*_SERCA_) as a function of internal Ca^2+^ at 6 Hz in the SHAM case (continuous grey line) and AB case (continuous black line). Fittings at 6 Hz are represented in the SHAM case (dashed grey line) and AB case (dashed black line).

**Figure 8:**
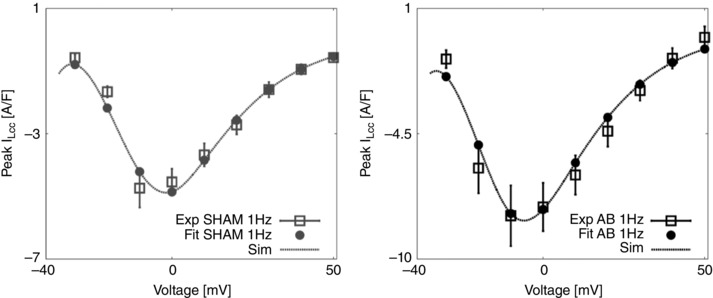
LCC fitting procedure and results Left, bell shaped peak *I*_LCC_–*V* relationship, experiments for the SHAM case at 1 Hz are represented as mean ± standard deviation (grey boxes) and fit (grey circles and dashed grey line). Right, bell shaped peak *I*_LCC_–*V* relationship, experiments for the AB case at 1 Hz are represented as mean ± standard deviation (black boxes) and fit (black circles and dashed black line).

Parameters for NCX, PMCa, SERCA, LCC and background Ca^2+^ were refitted as in Gattoni *et al*. (2016) with fitted results for NCX, SERCA and LCC shown in Figs 6, 7 and 8, respectively.

**Figure 9:**
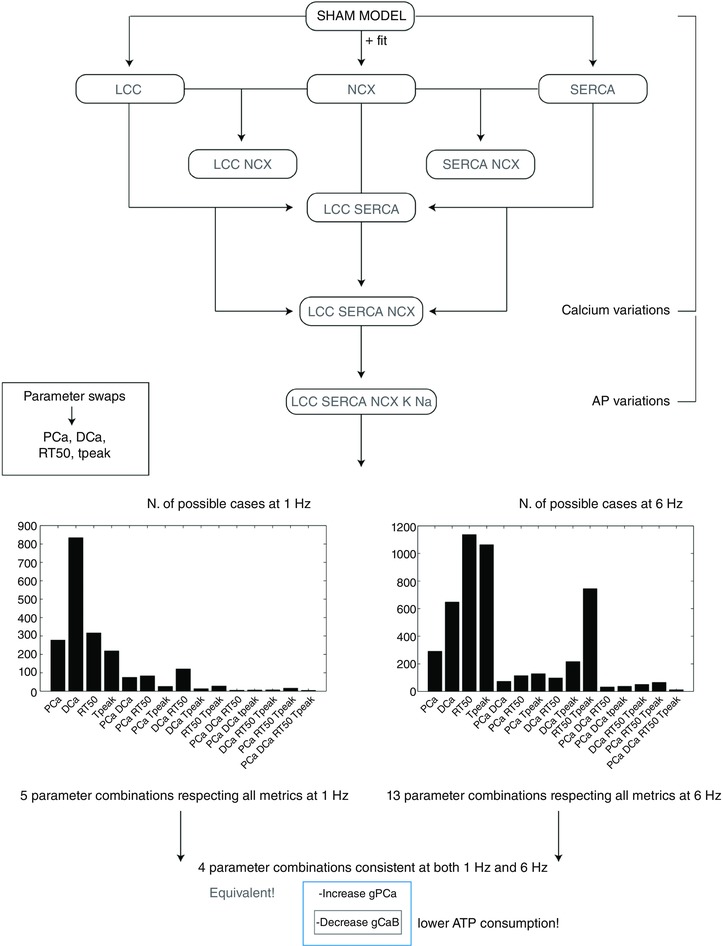
Schematic representation of the validation method Starting from the SHAM model at 1 Hz we have developed nine models by introducing single fittings for LCC, SERCA and NCX (3 models), combination of those (4 models) and all the fittings plus changes in K^+^ and Na^+^ channels (1 model) and K^+^, Na^+^ and CaB channels (1 model). For each of the nine models we have then performed parameter swaps of the most important protein parameters: *g*_SERCA_, *K*_SERCA_, *J*_L_, *J*_R_, *g*_CaB_, *g*_SRl_, *g*_t_, *g*_NCX_, *g*_pCa_, *g*_BK_, *g*_BNa_, *g*_K1_, *g*_Na_, *g*_ss_, NaK_max_, *B*_CMDN_ and *B*_TRPN_. The same process was repeated at 6 Hz. All developed models, representing 5814 parameter combinations (18 models × 17 parameters × 19 values) were run to steady state. For each model we have recorded PCa, DCa, RT_50_ and *T*_peak_. Ultimately, we have observed the number of combinations for which simulated PCa, DCa, RT_50_ and DCa fall within 20% variability of those four experimentally measured metrics. [Color figure can be viewed at wileyonlinelibrary.com]

Specific fittings of *I*_to_, *I*_K1_, *I*_ss_ and *I*_Na_ to account for AB AP phenotypes are described below.

#### K^+^ currents (*I*_to_, *I*_K1_, *I*_ss_)

Prolongation of the cardiac action potential is a feature of cardiac hypertrophy and has been previously reported in many studies including rat left ventricular myocardium following infarction (Qin *et al*. 1996; Rozanski *et al*. 1998), catecholamine‐induced rat hypertrophy (Meszaros *et al*. 1996), rat hypertrophy following aortic banding (Scamps *et al*. 1990) and human heart failure (Beuckelmann *et al*. 1992). The experimental studies we have performed in rats at 37**°**C revealed a major increase in APD in the AB case compared with the SHAM case at both 1 Hz and 6 Hz frequencies (as seen in Fig. 3 in the main text). Changes in Ca^2+^ dynamics alone did not allow such a degree of APD increase at either frequency suggesting that major changes in Na^+^ and K^+^ current could be responsible for this behaviour. Potential contributors to increased APD duration are the transient outward K^+^ current ($I_{to}$), the inwardly rectifying K^+^ current ($I_{K1}$) and the steady state K^+^ current ($I_{ss}$), all playing a significant role in rat repolarization (Amin *et al*. 2010; Chae *et al*. 2012). A decrease in repolarizing K^+^ currents has been previously reported in cells from failing human hearts (Beuckelmann *et al*. 1992) and rat models of heart disease (Scamps *et al*. 1990; Meszaros *et al*. 1996; Qin *et al*. 1996; Wickenden *et al*. 1997; Rozanski *et al*. 1998). Kaprielian *et al*. (Kaprielian *et al*. 1999) showed a decrease in $I_{to}$ current of about 45% in rats myocytes following aortic banding compared with myocytes from sham‐operated rats. A similar result was obtained by Benitah *et al*. (Bénitah *et al*. 1993) and Tomita *et al*. (Tomita *et al*. 1994) in rats following AB. A reduction in $I_{to}$ current was also shown by Qin *et al*. in rats following coronary artery ligation (Qin *et al*. 1996) and Meszaros *et al*. (Meszaros *et al*. 1996) who performed studies in rat with catecholamine‐induced cardiac hypertrophy and showed unchanged kinetics but 50% decrease in $I_{to}$ current, thought to be responsible for prolonged APD. A reduction of the $I_{K1}$ current during the development of hypertrophy and HF was reported in many animal studies. Among those, Kaprielian *et al*. (Kaprielian *et al*. 1999) showed a decrease in $I_{K1}$ current of about 25% in rats with aortic banding and the same finding was supported by Qin *et al*. (Qin *et al*. 1996). Fauconnier *et al*. (Fauconnier *et al*. 2005) showed a reduction of the $I_{K1}$ current in rats with HF. Reduced $I_{K1}$ activity was also shown in failing human hearts (Beuckelmann *et al*. 1992) and in rabbits with hypertrophy where Pogwidz *et al*. measured a 49% decrease in the $I_{K1}$ current. The steady‐state K^+^ current $I_{ss}$ was also reported to be slightly decreased in rats following aortic banding (Kaprielian *et al*. 1999). Following the results reported in the literature, in our model we have decreased the $I_{to}$, $I_{K1}$ and $I_{ss}$ currents by reducing the correspondent conductivities (see Table 5), in order to obtain prolonged APD and simulated Ca^2+^ transient as close as possible to the experimental Ca^2+^ transients.

#### Intracellular Na^+^ current (*I*_Na_)

Previous studies reported a reduction in the intracellular Na^+^ current $I_{Na}$ in rats (Nand & Doggrell, 2000), dogs (Maltsev *et al*. 2002) and human (Hasenfuss *et al*. 1999; Pieske, 2002). We reduced the $I_{Na}$ conductivity parameter in the AB model to achieve the peak of the AP at 1 Hz (see Table 5).

### Validation method and tables

**Figure 10:**
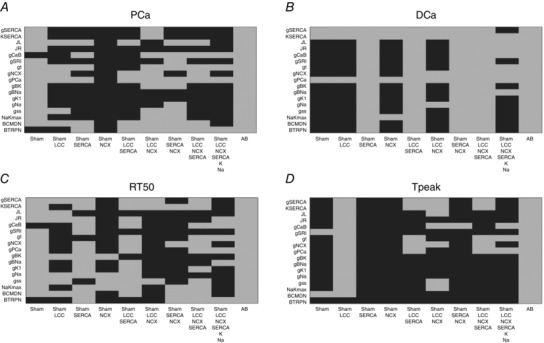
Single metrics at 1 Hz Parameters combinations falling inside (grey) and outside (black) single metrics experimental ranges. *A*, possible combinations of parameters falling inside a 20% variability range from PCa experimental measurements (grey). *B*, possible combinations of parameters falling inside a 20% variability range from DCa experimental measurements (grey). *C*, possible combinations of parameters falling inside a 20% variability range from RT_50_ experimental measurements (grey). *D*, possible combinations of parameters falling inside a 20% variability range from *T*_peak_ experimental measurements (grey).

**Figure 11:**
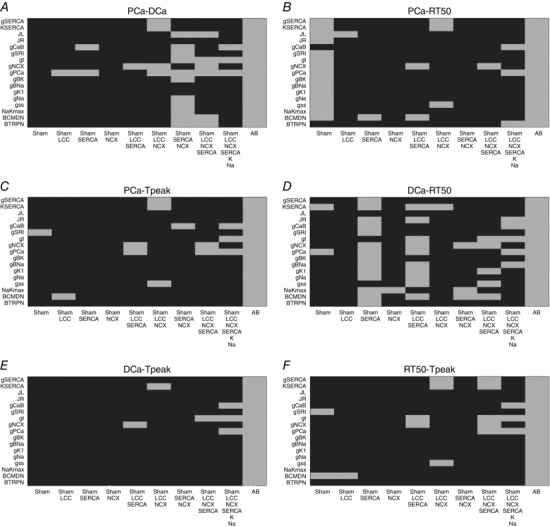
Metric couples at 1 Hz Parameters combinations falling inside (grey) and outside (black) experimental ranges for two metrics. *A*, possible combinations of parameters falling inside a 20% variability range from PCa and DCa experimental measurements (grey). *B*, possible combinations of parameters falling inside a 20% variability range from PCa and RT_50_ experimental measurements (grey). *C*, possible combinations of parameters falling inside a 20% variability range from PCa and *T*_peak_ experimental measurements (grey). *D*, possible combinations of parameters falling inside a 20% variability range from DCa and RT_50_ experimental measurements (grey). *E*, possible combinations of parameters falling inside a 20% variability range from DCa and *T*_peak_ experimental measurements (grey). *F*, possible combinations of parameters falling inside a 20% variability range from RT_50_ and T experimental measurements (grey).

**Figure 12:**
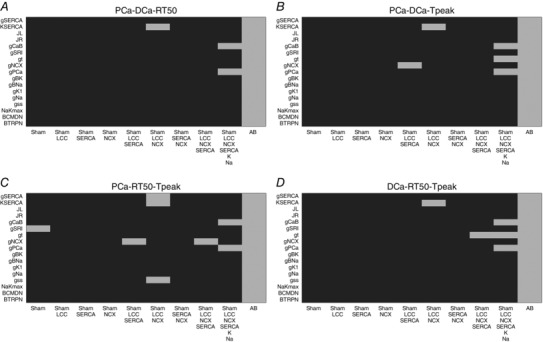
Metric triplets at 1 Hz Parameters combinations falling inside (grey) and outside (black) experimental ranges for three metrics. *A*, possible combinations of parameters falling inside a 20% variability range from PCa, DCa and RT_50_ experimental measurements (grey). *B*, possible combinations of parameters falling inside a 20% variability range from PCa DCa and *T*_peak_ experimental measurements (grey). *C*, possible combinations of parameters falling inside a 20% variability range from PCa, RT_50_ and *T*_peak_ experimental measurements (grey). *D*, possible combinations of parameters falling inside a 20% variability range from DCa, RT_50_ and *T*_peak_ experimental measurements (grey).

We have developed a validation method by exploring nine model variants (LCC, NCX, SERCA, LCC–SERCA, NCX–SERCA, LCC–NCX, LCC–SERCA–NCX, LCC–SERCA–NCX–K–Na and AB), obtained through permutation of parameters. For each simulation we have evaluated four metrics: peak Ca^2+^ (PCa), diastolic Ca^2+^ (DCa), relaxation time of the Ca^2+^ transient at 50% decay (RT_50_) and time to peak of the Ca^2+^ transient (*T*_peak_). For each box in the tables we have performed 19 different simulations (reproducing the 19 perturbed values for each parameter). The box is coloured in grey if at least one of those 19 simulations falls inside a 20% variability of the metric AB experimental range. For example, if we focus on the top left table in Fig. 10, we are looking at the results for one singe metric PCa. The top left box of this table is grey. In this specific case we have run 19 different simulations starting from the SHAM model (column) by perturbing the parameter *g*_SERCA_ (row) and in at least one of these 19 simulations we have found a PCa value falling inside a 20% variability of the PCa metric AB experimental range. Trivially, all the boxes for the AB model will be grey, as we already know that there is at least one PCa value falling inside a 20% variability of the PCa metric AB experimental range and that corresponds with the parameter value chosen in our AB model. It must be noted that, when considering more than one Ca^2+^ phenotype in Figs 11, 12, 14, 15 and 16, the box is coloured in grey if at least one of the 19 simulations falls inside a 20% variability of the considered phenotypes AB experimental range at the same parameter value. In this way, Fig. 16 shows that the AB models are able to replicate all four key phenotypes used to characterize cellular Ca^2+^ handling at both 1 Hz and 6 Hz.

**Figure 13:**
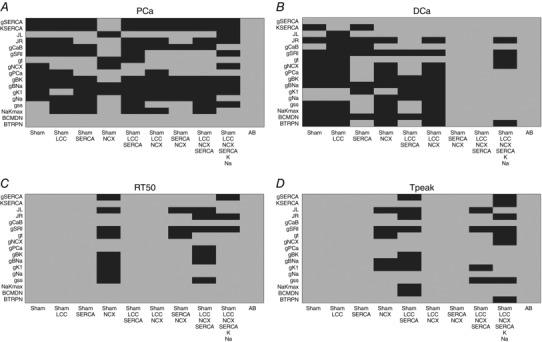
Single metrics at 6 Hz Parameters combinations falling inside (grey) and outside (black) single metrics experimental ranges. *A*, possible combinations of parameters falling inside a 20% variability range from PCa experimental measurements (grey). *B*, possible combinations of parameters falling inside a 20% variability range from DCa experimental measurements (grey). *C*, possible combinations of parameters falling inside a 20% variability range from RT_50_ experimental measurements (grey). *D*, possible combinations of parameters falling inside a 20% variability range from *T*_peak_ experimental measurements (grey).

**Figure 14:**
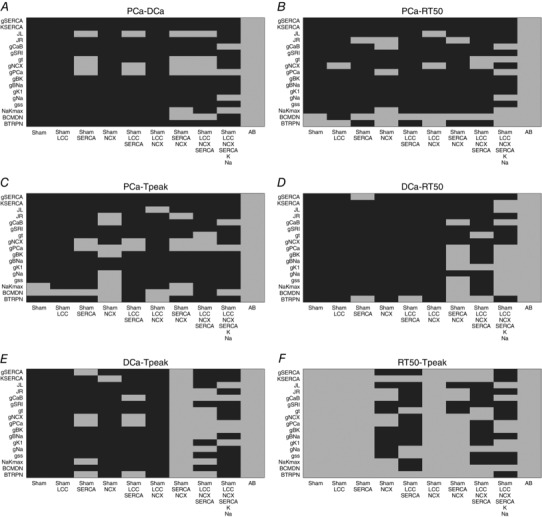
Metric couples at 6 Hz Parameters combinations falling inside (grey) and outside (black) experimental ranges for two metrics. *A*, possible combinations of parameters falling inside a 20% variability range from PCa and DCa experimental measurements (grey). *B*, possible combinations of parameters falling inside a 20% variability range from PCa and RT_50_ experimental measurements (grey). *C*, possible combinations of parameters falling inside a 20% variability range from PCa and *T*_peak_ experimental measurements (grey). *D*, possible combinations of parameters falling inside a 20% variability range from DCa and RT_50_ experimental measurements (grey). *E*, possible combinations of parameters falling inside a 20% variability range from DCa and *T*_peak_ experimental measurements (grey). *F*, possible combinations of parameters falling inside a 20% variability range from RT_50_ and *T*_peak_ experimental measurements (grey).

**Figure 15:**
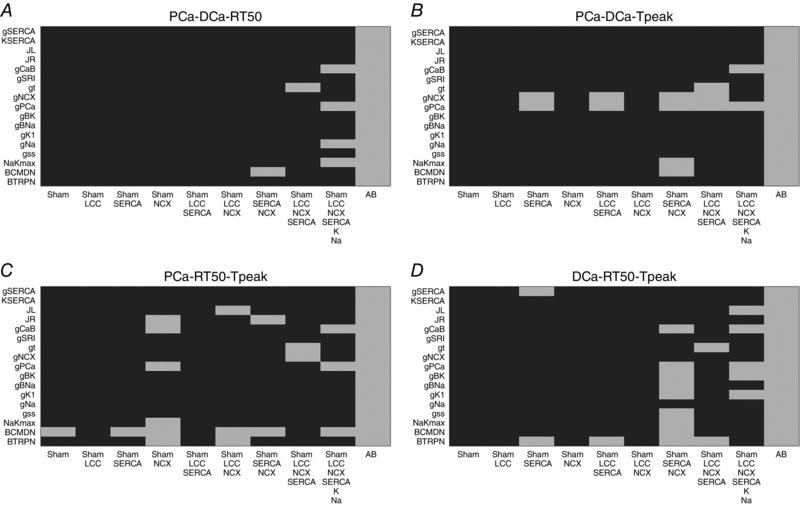
Metric triplets at 6 Hz Parameters combinations falling inside (grey) and outside (black) experimental ranges for three metrics. *A*, possible combinations of parameters falling inside a 20% variability range from PCa, DCa and RT_50_ experimental measurements (grey). *B*, possible combinations of parameters falling inside a 20% variability range from PCa DCa and *T*_peak_ experimental measurements (grey). *C*, possible combinations of parameters falling inside a 20% variability range from PCa, RT_50_ and *T*_peak_ experimental measurements (grey). *D*, possible combinations of parameters falling inside a 20% variability range from DCa, RT_50_ and *T*_peak_ experimental measurements (grey).

**Figure 16:**
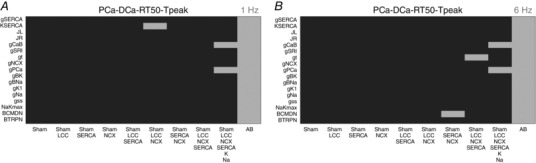
All metrics at 6 Hz Parameters combinations falling inside (grey) and outside (black) all metrics experimental ranges. *A*, possible combinations of parameters falling inside a 20% variability range from PCa, DCa, RT_50_ and *T*_peak_ experimental measurements at 1 Hz (grey). *B*, possible combinations of parameters falling inside a 20% variability range from PCa, DCa, RT_50_ and *T*_peak_ experimental measurements at 6 Hz (grey).

Among the 5814 combinations tested in the validation process only four parameter combinations are able to match all four phenotypes at both pacing frequencies and correspond to an increase in Ca^2+^ pump conductivity *g*_PCa_ $\;\varepsilon \;[1.256\times \;10^{-5},\;1.99\times \;10^{-5}]$ and a decrease in the background current conductivity *g*_CaB_ $\varepsilon \;[0.5024\times 10^{-8},\;1.0024\times \;10^{-8}]$. In Table 2 we show the resulting PCa, DCa, RT_50_, *T*_peak_ and Ca_amp_ values for those four parameter combinations.

### Limitations and future directions

The developed model reveals a number of limitations that were already discussed in Gattoni *et al*. (2016). No experimental data were available regarding the behaviour of the Na^+^ and K^+^ channels in both the SHAM and AB conditions. Collection of new experimental data on Na^+^ and K^+^ channels and their changes with frequency and temperature will certainly help to make the model more reliable.

Alternative Ca^2+^ regulators and pathways may also be important. In particular late Na^+^, store‐operated calcium entry (SOCE) and transient receptor potential (TRP) channels might influence both systolic and diastolic calcium concentrations, and we did not have sufficient data to constrain these potential contributors to Ca^2+^ regulation. However, recent studies have shown that SOCE only appears in about 30% of hypertrophied rat myocytes and are absent from adult cardiomyocytes (Luo *et al*. 2012) while TRP channels are indicated as having a role in Ca^2+^ dynamics but through manipulating Ca^2+^ regulation as opposed to acting as a Ca^2+^ pathway (Eder & Molkentin, 2011). Furthermore, the model we developed is not a classic systolic heart failure model, being based on data acquired during concentric hypertrophy, a condition under which there is much less known about the influence of late Na^+^, SOCE and TRP channels. The current data available are unable to quantify the possible effect by these alternative Ca^2+^ regulators and pathways and their role under conditions of concentric hypertrophy cannot be ruled out. It is also worth noting that, due to the significant differences in calcium handling between rats and mice compared with other mammalian species, the model predictions about the roles of LCC and SERCA may not hold true in other species.

The model was fitted to representative traces. In Table 1 the peak Ca^2+^ at 1 Hz in the representative traces decreased by 10% between the control and the AB rats, while the model predicts an increase of 2.7%. This is caused by an elevated prediction of the peak Ca^2+^ in the AB model. While the model does not capture the small decrease at 1 Hz, the much larger (40%) decrease in peak Ca^2+^ at the more physiological 6 Hz is captured and the model replicates the overall Ca^2+^ transient shape, as shown in Fig. 1.

Furthermore, in our model we have reported an increase in the SR load in the AB case compared with the SHAM case, consistent with an increase in SERCA activity reported both experimentally and in our simulations. In their paper, Røe *et al*. (2017) reported a decreased magnitude of the caffeine‐induced Ca^2+^ transient suggesting a decreased SR load. A decrease in SR load, despite an increase in SERCA, could be explained by a sustained elevated leak from the SR. However, we had no recordings to support this.

Although the choice of 20% variability ranges in the validation process can also be seen as a limitation in our model, it was made due to the high variability of data and our Ca^2+^ results were shown to be good approximations of the experimental results.

In our work, the effects of AB were only assessed 6 weeks after ligation of the aorta. As a feature work, it will be interesting to observe the temporal changes during the development of diastolic dysfunction following AB. In particular, the results of our work have highlighted SERCA as a possible marker of the transition from a compensatory to a decompensatory state; experimental data at 12 weeks will be helpful to confirm or reject this hypothesis.

## Additional information

### Competing interests

The authors declare there are no competing interests.

### Author contributions

S.G. re‐fitted the model, performed the analyses and wrote the manuscript. A.T.R, J.M.A. and W.L. acquired the data and assisted in the writing of the manuscript. S.A.N. and N.P.S. contributed to the conception and motivation of the analysis and to the interpretation of the results, and assisted in the writing of the manuscript. All authors approved the final version of the manuscript. All authors agree to be accountable for all aspects of the work in ensuring that questions related to the accuracy or integrity of any part of the work are appropriately investigated and resolved. All persons designated as authors qualify for authorship.

### Funding

This work was supported by the Virtual Physiological Rat Project (NIH 1 P50 GM094503‐01). The project has also received funding from the European Union's Horizon 2020 research and innovation programme (Consolidator grant, WEL) under grant agreement No 647714, South‐Eastern Norway Regional Health Authority, Anders Jahre's Fund for the Promotion of Science, the Norwegian Institute of Public Health, Oslo University Hospital Ullevål, and the University of Oslo. The authors acknowledge financial support from the Department of Health via the National Institute for Health Research (NIHR) comprehensive Biomedical Research Centre award to Guy's and St Thomas’ NHS Foundation Trust in partnership with King's College London and King's College Hospital NHS Foundation Trust.
